# Alterations in Regional Homogeneity of Spontaneous Brain Activity in Late-Life Subthreshold Depression

**DOI:** 10.1371/journal.pone.0053148

**Published:** 2013-01-02

**Authors:** Zhenling Ma, Rui Li, Jing Yu, Yong He, Juan Li

**Affiliations:** 1 Center on Aging Psychology, Key Laboratory of Mental Health, Institute of Psychology, Chinese Academy of Sciences, Beijing, China; 2 School of Nursing, Peking Union Medical College, Beijing, China; 3 Magnetic Resonance Imaging Research Center, Institute of Psychology, Chinese Academy of Sciences, Beijing, China; 4 Research Center of Emotion Regulation, Beijing Normal University, Beijing, China; 5 School of Psychology, Southwest University, Chongqing, China; 6 State Key Laboratory of Cognitive Neuroscience and Learning, Beijing Normal University, Beijing, China; Hangzhou Normal University, China

## Abstract

The early detection of major depression in elderly individuals who are at risk of developing the disease is of prime importance when it comes to the prevention of geriatric depression. We used resting-state functional magnetic resonance imaging (fMRI) to examine changes in regional homogeneity (ReHo) of spontaneous activity in late-life subthreshold depression (StD), and we evaluated the sensitivity/specificity performance of these changes. Nineteen elderly individuals with StD and 18 elderly controls underwent a resting-state fMRI scan. The ReHo approach was employed to examine whether StD was related to alterations in resting-state neural activity, in the form of abnormal regional synchronization. Receiver operating characteristic curve analysis and the Fisher stepwise discriminant analysis were used to evaluate the sensitivity/specificity characteristics of the ReHo index in discriminating between the StD subjects and normal controls. The results demonstrated that, compared to controls, StD subjects display lower ReHo in the right orbitofrontal cortex (OFC), left dorsolateral prefrontal cortex (DLPFC), left postcentral gyrus (PCG), and left middle frontal and inferior temporal gyri, as well as higher ReHo in the bilateral insula and right DLPFC. The left PCG and the right DLPFC, OFC, and posterior insula, together reported a predictive accuracy of 91.9%. These results suggest that the regional activity coherence was changed in the resting brain of StD subjects, and that these alterations may serve as potential markers for the early detection of StD in late-life depression.

## Introduction

Depression is a common emotional and mental disability in the elderly population [1]. It is characterized by the presence of depressed mood, and the loss of interest or pleasure in daily activities, and other depression symptoms [2]. It has a serious effect on the quality of life of elderly individuals and increases their risk of developing physical diseases. Thus, its early detection in individuals who are at a risk of developing major depressive disorder (MDD) is of prime importance when it comes to the prevention of geriatric depression.

Neuroimaging methods have been widely employed to investigate the underpinnings of various neurological and psychiatric disorders [3], [4]. A significant trend in recent neuroimaging research is the increasing use of resting-state functional magnetic resonance imaging (fMRI) to study brain disorders [5], [6]. By examining the spontaneous fluctuations in the brain with resting-state fMRI, it is hoped that insight will be gained into the alterations that occur in the diseased brain, which in turn may lead to the discovery of neuroimaging markers that can detect brain disorders in the early stages [7], [8].

Functional neuroimaging studies of depression have attributed its incidence to widespread activity abnormalities in certain brain regions, including the prefrontal cortex, limbic areas, and subcortical structures [9]–[15]. In resting-state fMRI studies of depression, most of the current work has focused on MDD. Various studies have reported that MDD patients show alterations in regional activity, functional connectivity, and large-scale connectivity. Regional brain activity in the frontal, temporal, occipital, and cerebellar lobes, as well as in the thalamus and insula, displays reduced local synchronization [10], [16], [17]. Abnormal functional connectivity in MDD has been demonstrated by decreased pregenual anterior cingulate cortex (pACC)-dorsomedial thalamus connectivity [18], pACC-anterior insula connectivity [19], amygdala-anterior insula connectivity, and bilateral lingual gyrus connectivity [11], as well as increased connectivity between the subgenual cingulate cortex and thalamus [9]. In geriatric depression, altered functional connectivity with the caudate nuclei [20] and posterior cingulate cortex [21] has been reported. Furthermore, a recent large-scale connectivity study revealed that MDD could also disrupt the topological organization of functional brain networks [12].

Despite the increasing understanding of MDD, it is unclear if alterations in resting-state fluctuations occur in elderly individuals with subthreshold depression (StD). These individuals have symptoms of depression but do not meet the diagnostic criteria for MDD. Given that the incidence of MDD is highly increased in elderly individuals with subthreshold depression [22], we speculated that the resting-state brain activity of these individuals might have presented abnormalities at an earlier stage, prior to the incidence of disease. If alterations in resting-state brain activity do occur in elderly individuals at risk of developing MDD, it may aid in the early diagnosis of geriatric depression and may improve the quantitative evaluation of treatment that is aimed at the prevention of this disorder.

Specifically, the current study employed the regional homogeneity (ReHo) approach [23] to investigate if resting-state brain activity is altered in elderly individuals with StD. Based on the assumption that spatially neighboring voxels show similar temporal hemodynamic characteristics [24], ReHo employs Kendall’s coefficient of concordance (KCC) [25] to measure regional spontaneous activity coherence. An important advantage of using the ReHo method over other methods is that it can examine the regional activity characteristics of each voxel. It can also detect changes or modulations that are induced by different conditions across the whole brain in a voxel-by-voxel manner, without requiring any prior knowledge. It has been applied to a number of neurological and psychiatric studies and proven effective in detecting disease-associated regional activity changes [10], [26]–[28]. Since little is known about the resting-state brain activity of elderly individuals with StD, ReHo is particularly useful for exploratory analysis, which in turn could aid future in-depth studies, such as functional connectivity analysis.

Here, we hypothesized that regional coherence of spontaneous activity is altered in elderly individuals with StD. To test our hypothesis, we collected resting-state fMRI data from 19 subjects with StD and 18 normal controls (NC). We then used the ReHo method to measure any changes in regional coherence that may have been induced by StD. Correlation between ReHo measurements and self-rated measurements of depression was evaluated, and the sensitivity/specificity performance of the resting-state ReHo index in discriminating between StD subjects and NC subjects was assessed.

## Materials and Methods

### Subjects

The NC group had a mean age of 66.4 years, and consisted of 8 males and 10 females. The group of individuals with StD had a mean age of 66.5 years, and consisted of 7 males and 12 females. The two groups did not differ significantly in age (*p* = 0.96) or gender distribution (*p* = 0.64). The Center for Epidemiologic Studies Depression Scale (CES-D) was used to determine each subject’s depression quotient. The mean score for the StD group was 16.4, with a standard deviation (SD) of 4.9. The mean CES-D score for the NC group was 1.1, with a SD of 1.6. The StD subjects had a CES-D cutoff of ≥8, a Mini Mental State Examination (MMSE) cutoff of ≥24, and none of them met the DSM-IV diagnostic criteria for MDD. The NC subjects had a CES-D score ≤5 and a MMSE score ≥24. None of our subjects had cancer, a history of cerebrovascular disease, head injury, or mental illness. They were not taking antidepressants or other psychotropic medication during the period of this study. Five of them (1 NC subject and 4 StD subjects) were taking medication for hypertension; 3 of them (2 NC subjects and 1 StD subject) were taking medication for diabetes; and 1 StD subject was taking medication for coronary artery disease. The clinical and demographic characteristics of these subjects are shown in Table 1.

**Table 1:** Characteristics of the StD, and healthy controls.

Characteristics	StD	NC	*P* value
N (M/F)	19 (7/12)	18 (8/10)	0.64^a^
Age, years	66.5±5.7	66.4±3.9	0.96^b^
Education, years	13.2±2.7	13.5±2.8	0.75^b^
MMSE	28.3±1.6	28.8±1.5	0.26^b^
CES-D	16.4±4.9	1.1±1.6	<0.01^b^

a The *P* value for gender distribution was obtained by chi-square test.

b The *P* values were obtained by *t*-test.

### Ethics Statement

The purpose of this study was explained to all of the participants, and each of them gave written consent that was approved by the Research Ethics Committee of the Institute of Psychology, Chinese Academy of Sciences.

### Data Acquisition

A 3-T Siemens scanner, equipped for echo planar imaging (EPI), was used for image acquisition at the Imaging Center for Brain Research, Beijing Normal University. For each participant, 160 EPI functional volumes were collected. The following parameters were used: time repetition (TR) = 3000 ms, time echo (TE) = 30 ms, flip angle = 90°, field of view (FOV) = 200×200 mm^2^, 45 axial slices, thickness = 3.0 mm, gap = 0.6 mm, in-plane resolution = 64×64, and voxel size = 3.125×3.125×3.0 mm^3^. High-resolution, three-dimensional T1-weighted structural images were acquired for each subject, with the following parameters: 176 slices, resolution = 256×256, voxel size = 1×1×1 mm^3^, TR = 1900 ms, TE = 2.2 ms, and flip angle = 9°. During the scan, the subjects were instructed to keep their eyes closed and not to think of anything in particular.

### Data Analysis

Raw fMRI data was preprocessed. For each participant, the first 5 scans of the fMRI time series were discarded to allow for equilibration of the magnetic field. The remaining images were preprocessed using SPM8 (http://www.fil.ion.ucl.ac.uk/spm) and DPARSF V2.0 Basic Edition [29]. Data pre-processing steps included within-subject spatial realignment, slice timing correction, and between-subject spatial normalization to a standard brain template in the Montreal Neurological Institute (MNI) coordinate space. Following this, detrending and temporal band-pass filtering of the fMRI data was carried out in order to reduce the effects of low-frequency drifts and physiological high-frequency noise [29]. Unified segmentation algorithm [30] was performed on T1 images to obtain gray matter intensity maps, and these were then used as covariates in the between-group ReHo comparison analysis in order to rule out any potential influence of gray matter abnormalities [31], [32].

Across the whole brain, KCC [25] was used to measure the ReHo of the time series of a given voxel with its nearest 26 neighbors in a voxel-wise manner [23]. ReHo maps were first constructed for each subject by calculating the KCC of each voxel within the entire resting brain, and in order to reduce the effect of individual variability, each ReHo map was then divided by the mean ReHo of the whole brain. Finally, the ReHo maps were spatially smoothed with a 4-mm Gaussian filter to decrease spatial noise. To establish the within-group ReHo maps voxel-wise, a one-sample *t*-test was performed on the individual ReHo maps for each group. To determine the between-group ReHo differences, a two-sample *t*-test was performed on the individual ReHo maps of the two groups, by taking voxel-wise gray matter volumes as covariates [31], [32].

Based on these ReHo findings, we identified several brain regions that demonstrated significant between-group differences, which we classified as regions of interest (ROIs). These ROIs were then examined for their correlation with the CES-D, and evaluated for their sensitivity/specificity characteristics in distinguishing the two groups. Each ROI was defined as a sphere centered at the voxel showing the highest statistical difference, with a radius of 6 mm, and only the voxels exceeding the threshold in the two-sample *t*-test ReHo comparison map were included in the ROIs. For each ROI, the mean ReHo value was extracted by averaging the ReHo values over all of the voxels within the ROI for each individual subject.

To investigate the relationship between CES-D and resting-state ReHo, partial correlation analysis was performed for each ROI’s ReHo and CES-D in StD Group. In order to identify effective biomarkers that can distinguish between StD subjects and NC subjects, receiver operating characteristic (ROC) curve analysis was used to summarize the sensitivity/specificity characteristics of each ROI, and the optimal cut-off ReHo values for these ROIs was determined. In addition, Fisher stepwise discriminant analysis was used to predict the state of each subject, according to the ReHo characteristics of all the ROIs. The discriminant analysis was cross-validated using the leave-one-out method. The sensitivity and specificity values, as well as the mean classification accuracy, were calculated to show the predictive power of these ROIs during the classification process.

## Results

### Resting-state ReHo Maps for the NC and StD Groups

**Figure 1:**
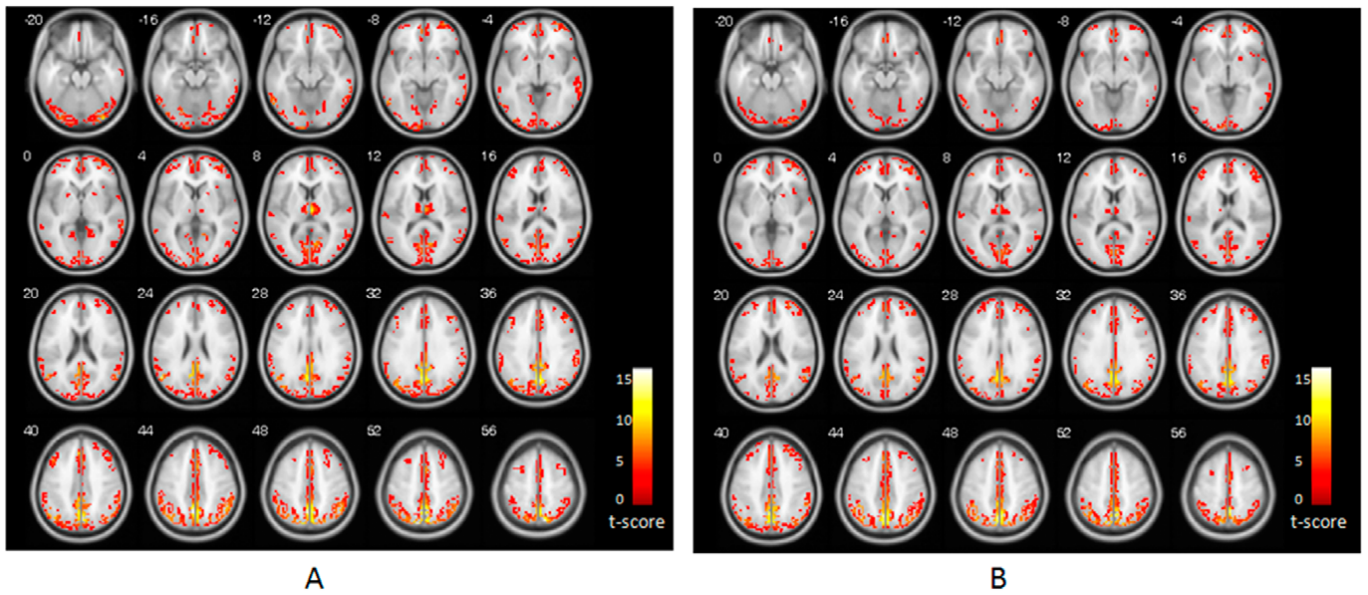
Results of ReHo shown as a KCC maps. (*A*) ReHo map for control group. (*B*) ReHo map for StD group. The numbers in the upper left of each image show the z-plane coordinate of the Montreal Neurological Institute (MNI) space. T-score bar is show at right (one sample *t*-test, thresholded at *p*<0.05, corrected by FDR with an extent threshold 5 contiguous voxels).

Figure 1 shows the one-sample *t*-test result (corrected by false discovery rate [FDR]; p<0.05) of our ReHo analysis in NC subjects (Figure 1*A*) and StD subjects (Figure 1*B*). Consistent with previous studies, ReHo maps for both NC and StD subjects displayed very similar spatial patterns to the default-mode network. The regions that demonstrated significantly higher ReHo included the precuneus/posterior cingulate cortex (PCC); medial, dorsolateral, and orbital prefrontal cortex; inferior, middle, and superior frontal gyrus; inferior, middle, and superior temporal gyrus; inferior and superior parietal lobule; thalamus; insula; pre- and postcentral gyrus, and putamen.

### Between-group ReHo Comparison

Brain regions demonstrating significant differences in ReHo between the two groups were detected by a two-sample *t*-test (*p*<0.005, uncorrected).

**Figure 2:**
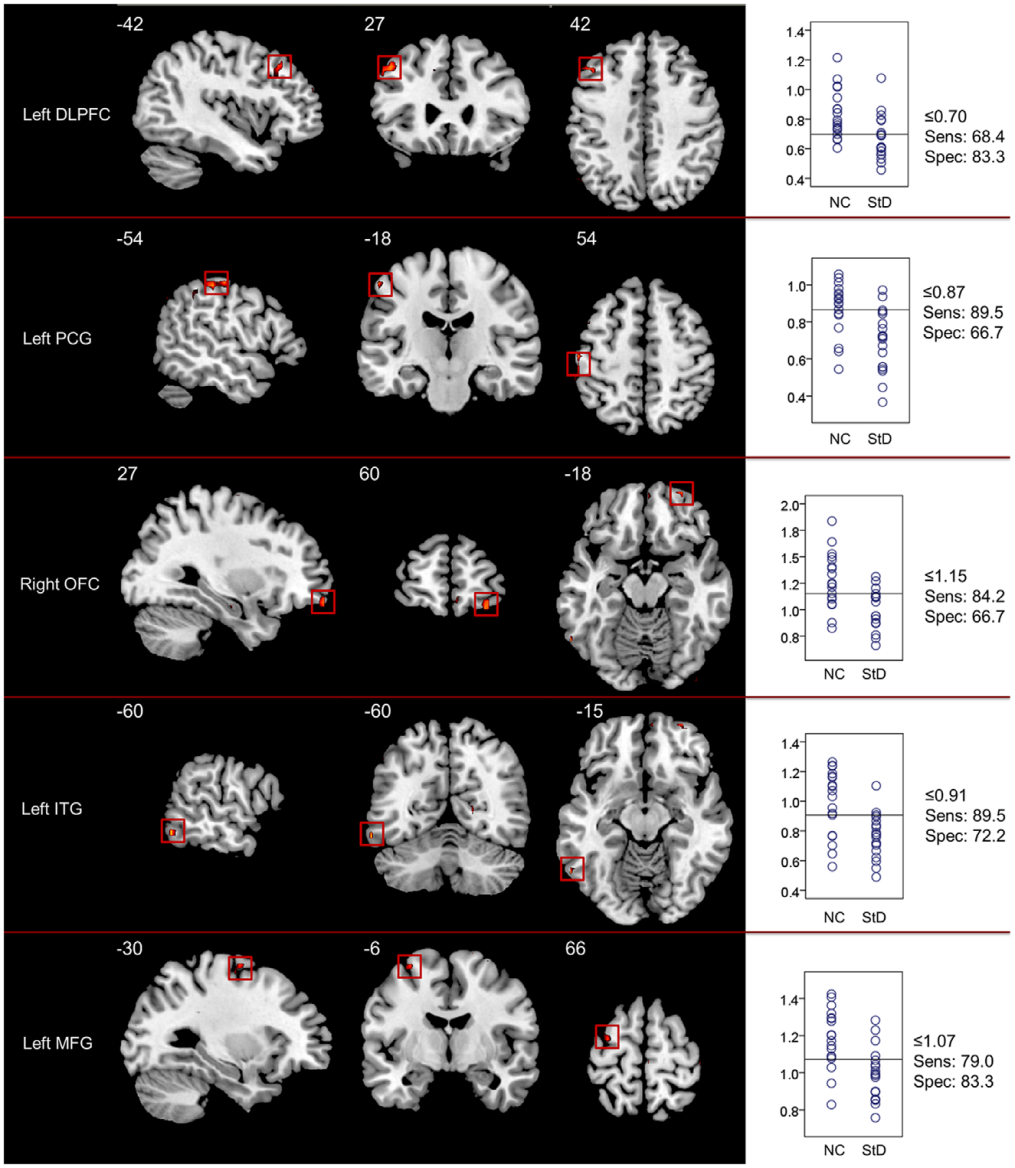
Brain regions (ROIs) showing significantly increased ReHo in NC (decreased ReHo in individuals with StD) (two sample *t*-test, thresholded at *p*<0.005 with an extent threshold 5 contiguous voxels, uncorrected) and their sensitivity/specificity plots. The left panel demonstrates the coronal, sagittal, and axial views of each ROI with the MNI location shown at the lower right. The right panel presents correspondingly the scatter plot for the ReHo in the region. Y-axis of the scatter plot is the extracted mean ReHo values, and the horizontal lines in the scatter plots are the cutpoints with the best separation between NC and StD that are evaluated from the ROC analysis. The sensitivity (Sens) and the specificity (Spec) at the cutpoints are shown at the right side of the scatter plots.

Compared to controls, StD subjects showed decreased ReHo in the right orbitofrontal cortex (OFC; Brodmann’s area [BA] 11), left middle frontal gyrus (MFG; BA 6), left dorsolateral prefrontal cortex (DLPFC; BA 8/9), left inferior temporal gyrus (ITG; BA 37), and left postcentral gyrus (PCG; BA 2) (Figure 2).

**Figure 3:**
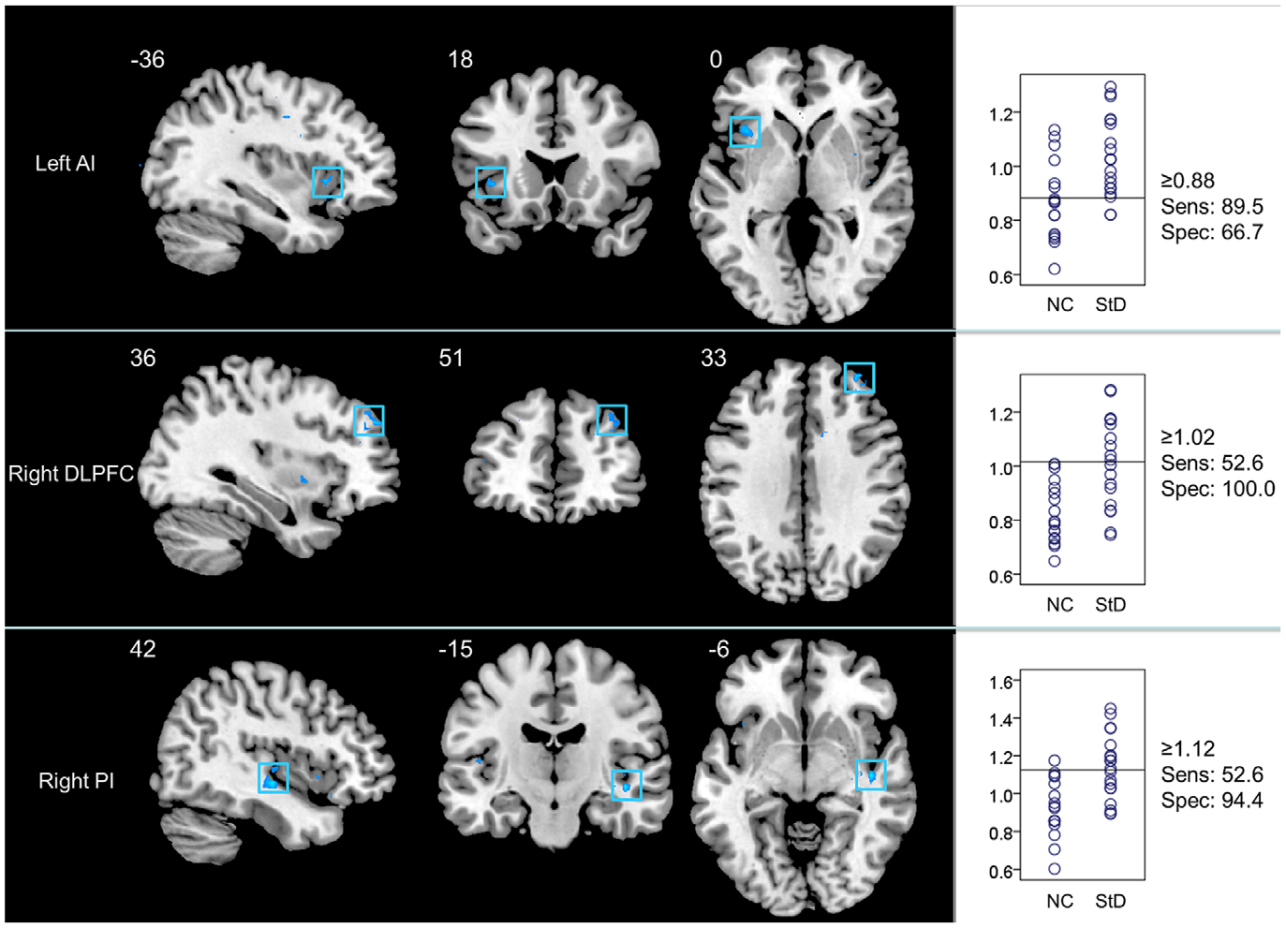
Brain regions (ROIs) showing significantly increased ReHo in StD group (decreased ReHo in NC) (two sample *t*-test, thresholded at *p*<0.005 with an extent threshold 5 contiguous voxels, uncorrected) and their sensitivity/specificity plots. The left panel demonstrates the coronal, sagittal, and axial views of each ROI with the MNI location shown at the lower right. The right panel presents correspondingly the scatter plot for the ReHo in the region. Annotations in the figures are identical with those in Figure 2.

StD subjects also showed increased ReHo in the left anterior insula (AI; BA13), right posterior insula (PI; BA 13), and right DLPFC (BA 9) (Figure 3).

Table 2 displays a detailed list of all regions that showed significant between-group differences. The eight regions with significant between-group differences were defined as the ROIs for the subsequent analysis.

**Table 2:** Brain regions showing significant between-group differences in ReHo.

Regions	L/R	BA	MNI location			Maximal t-value	Cluster size (voxels)
			x	y	z		
Inferior temporal gyrus (ITG)	L	37	−60	−60	−12	4.01	6
Dorsolateral prefrontal cortex (DLPFC)	L	8/9	−45	27	42	3.65	16
Postcentral gyrus (PCG)	L	2	−54	−27	51	3.56	14
Middle frontal gyrus (MFG)	L	6	−30	−6	66	3.16	8
Orbitofrontal cortex (OFC)	R	11	30	60	−18	3.50	9
Anterior insula (AI)	L	13	−39	21	0	3.39	10
Posterior insula (PI)	R	13	42	−15	−6	4.37	7
Dorsolateral prefrontal cortex (DLPFC)	R	9	33	51	33	3.93	10

L, left; R, right; BA, Brodmann area.

### Correlation between the ROI’s ReHo and CES-D

No significant correlation between CES-D scores and ReHo values were found in any of the eight regions (*p*>0.05).

### Sensitivity and Specificity Measures of ROI’s ReHo

As demonstrated in Figure 2 and Figure 3, among the 5 ROIs that showed decreased ReHo in StD subjects, the left ITG and the left MFG showed higher sensitivity and specificity (89.5% and 79.0% for sensitivity, and 72.2% and 83.3% for specificity), respectively. The left PCG and right OFC showed higher sensitivity (89.5% and 84.2%, respectively) but lower specificity (66.7% for both), while the left DLPFC had a higher specificity (83.3%) but a lower sensitivity (68.4%). Among the three regions that showed increased ReHo in StD subjects, the left AI showed higher sensitivity (89.5%) and lower specificity (66.7%), while the right PI and right DLPFC showed higher specificity (94.4% and 100.0%, respectively) and lower sensitivity (52.6% for both).

The eight ROIs that showed altered ReHo between the two groups were subjected to the Fisher stepwise discriminant analysis, using Wilk’s Lambda as the chosen selection criterion. The left PCG, right OFC, right DLPFC, and the right PI, were then selected as the principle variables to discriminate between StD subjects and NC subjects. Leave-one-out cross validation showed that the mean predictive accuracy was 91.9%, with a sensitivity of 94.7% and a specificity of 88.9%.

## Discussion

By applying the ReHo approach to the resting-state fMRI data of both the NC and StD groups, we found that a significantly different ReHo existed between the groups in some brain regions, suggesting that local resting-state activity coherence is altered in individuals with StD. Compared with NC subjects, those with StD displayed decreased ReHo in several clusters, including the right OFC, left MFG, left PCG, and left ITG. Increased ReHo in StD subjects was found in the bilateral insula. Two regional clusters in the left and right DLPFC demonstrated decreased and increased ReHo in StD subjects compared with NC subjects, respectively. These findings provide primary evidence of abnormal spontaneous activity in elderly individuals at a risk of developing MDD. In addition, we evaluated the sensitivity and specificity characteristics of the ROIs that showed between-group ReHo differences. Some separate regions such as left ITG and MFG were relatively sensitive and specific to StD in resting-state ReHo. Fisher stepwise discriminant analysis found that ReHo in the ROIs could predict the individual depressive state of the 37 subjects with a mean accuracy of 91.9%.

### Alterations in the Resting-state ReHo of StD Subjects

The finding of altered ReHo in the resting-state activity of StD subjects relative to NC subjects indicates that depression could affect neural activity early, when the individuals are still in the “at-risk” period. Altered resting-state activity has been reported primarily in clinical depression, especially in MDD. To the best of our knowledge, this is the first time that alterations in spontaneous activity in elderly individuals with StD have been reported. However, the alterations in ReHo that we found in the resting-state activity of StD subjects are not completely consistent with those found in clinical depression [10], [17]. Amongst the clusters that demonstrated between-group ReHo differences, the OFC and ITG have previously been demonstrated to show decreased ReHo in MDD; however, the insular regions also showed decreased ReHo in these patients [10], [16], [17]. The regional clusters found in the DLPFC, PCG, and MFG have previously been reported to show alterations in metabolism, structure, resting-state functional connectivity, and activation [15], [20], [34], [35].

It has previously been reported that the prefrontal cortex is an important brain region associated with symptoms of depression [8], [14], [15], [36], [37]. In our study, several regional clusters from the frontal cortex, including the left OFC, bilateral DLPFC, and left MFG, showed abnormal ReHo in StD subjects compared with NC subjects. This suggests that alterations in regional spontaneous activity exist in the prefrontal cortex of elderly individuals with StD, who have not yet been clinically diagnosed with depression.

The OFC is considered to play a major role in the pathophysiology of mood disorders, including depression [38], since it displays reductions in cortical thickness [39]–[41] and abnormalities in hemodynamic responses to emotional processing [42] in depressed patients. In late-life depression, the OFC also shows abnormally reduced grey matter volume [43]. In addition, reduced metabolism and cerebral blood flow (CBF) in the OFC are characteristics that differentiate between depressed and non-depressed patients with Parkinson’s disease [44]. In a resting-state fMRI study consisting of 22 MDD patients, with a mean age of 38.2 years [17], reduced regional coherence in the right OFC was found. The findings of this study, taken with those of previous reports on OFC, indicate that abnormal resting-state activity in this brain area may be an important marker for late-life depression.

A regional cluster in the left MFG, that was from the premotor cortex (BA 6), also demonstrated decreased ReHo in StD subjects. Abnormal activity in the premotor cortex has previously been suggested to account for the psychomotor retardation that is commonly observed in depressed patients [45]. A recent study by Walther et al. (2012) investigated the pathobiology of motor retardation in 22 MDD subjects who had a mean age of 44.0 years. They reported that the MFG (BA 6) showed decreased activity in the MDD subjects compared with controls, and that the correlation between resting-state CBF and motor performance could differentiate between the subjects with MDD and NC [35]. Although behavioral measurements did not show any obvious psychomotor retardation in our subjects with StD, the abnormal ReHo found in this area may imply that the brain has begun to change prior to the presence of obvious abnormal behavioral performances. The change in resting-state activity in this area, as well as its association with motor performance, needs to be investigated as the disease progresses from the “at-risk” stage to clinical depression.

Two regional clusters from the DLPFC showed significant differences in regional activity coherence. Lower ReHo in the left DLPFC and higher ReHo in the right DLPFC were observed in subjects with StD. These paradoxical changes in resting-state ReHo in the bilateral DLPFC support the asymmetry hypothesis of prefrontal activity in depression [33], [46], [47]. Previous positron emission tomography (PET) studies have shown lower CBF and metabolism in the left DLPFC, and higher CBF and metabolism in the right DLPFC in MDD [15], [48]. Additionally, fMRI studies of MDD have demonstrated altered activity in the left and right DLPFC. In an fMRI study of negative emotional judgment, Grimm et al. (2008) found imbalanced left-right DLPFC activity in patients with MDD [33]. It has been suggested that the left and right DLPFC are responsible for emotional judgment and the anticipation of emotional judgment, respectively [49], [50], and that they are involved separately in positive and negative emotional processing [51], [52]. We speculate that the paradoxical changes in resting-state regional coherence in the bilateral DLPFC might be an intrinsic expression of negative emotional bias or emotion regulation in these individuals, and that these paradoxical ReHo measurements might reflect an interesting biological marker for the characterization of elderly individuals at risk of developing depression.

In addition to decreased ReHo in the prefrontal cortex, regional clusters in the left PCG and ITG also showed reduced ReHo in StD subjects. Recent studies have shown that the PCG demonstrates altered resting-state functional connectivity in late-life depression [20] and reduced gray matter volume in remitting recurrent depressed patients [53]. It has also been reported that the ITG demonstrates structural [21], [54] and functional abnormalities, which correlate with self-reported anxiety [34] in depression. In this study, the alterations in regional activity coherence in these areas of the StD subjects might also reflect an important characterizing feature of brain activity alterations that occur in late-life individuals at risk of developing MDD.

Interestingly, two clusters from the bilateral insula demonstrated higher ReHo in StD subjects. The involvement of the insula in depression has been reported in previous PET and fMRI studies [55], [20]. It has been demonstrated to show decreased gray and white matter volume [55], and increased resting-state functional connectivity with the caudate nuclei [23] in patients with late-life depression. However, in studies by Peng et al. (2011) [16] and Yao et al. (2009) [17] the insula showed decreased resting-state ReHo in MDD patients. It is possible that the differences observed were caused by discrepancies between experimental subjects. Previous studies of ReHo have concentrated on MDD patients, but the current work focused on individuals at the StD stage. The paradoxical findings that have been observed in the insula suggests that alterations in brain activity that occur at the “at-risk” stage and at the clinical depression stage do not follow a simple pattern, such as a linear process of gradually increasing or decreasing brain activity, but rather that it occurs in a more complicated manner. In addition, differences that existed in subject age are important. The present study was restricted to elderly individuals, while the 16 MDD subjects in the study by Peng et al. (2011) ranged in age from 20 to 50 years [16], and 22 MDD subjects in the study by Yao et al. (2009) had a mean age of 38.2 years [17].

### Evaluation of Resting-state ReHo-based Biomarkers

Although no significant correlations were found between CES-D scores and ReHo values in StD subjects, the ROC and the discriminant analysis demonstrated that ReHo values in these ROIs performed well in differentiating between the two groups. ROC analysis for each of the eight ROIs that showed significant between-group differences in regional coherence found that some of them, including ROIs from the left ITG and MFG, had sensitivities and specificities more than 70%. The other regions, including the left DLPFC, right PI, and right DLPFC, had higher specificities (83.3%, 94.4%, and 100.0%, respectively), but lower sensitivities (68.4%, 52.6%, and 52.6%, respectively). Other regions, including the left PCG and left AI, showed higher sensitivities (89.5% for both) but lower specificities (66.7% for both). These results suggest that the sensitivity/specificity characteristic of resting-state ReHo varies among the different ROIs. Given that the Fisher stepwise discriminant analysis reported a mean predictive accuracy of 91.9%, with sensitivity of 94.7% and specificity of 88.9%, ReHo in these ROIs should be used in future large-sample studies to evaluate its performance. It seems promising that ReHo measurements in these regions could be used as neuroimaging biomarkers in the future for characterizing StD in elderly individuals.

### Limitations of the Current Study

Several limitations of the present study deserve mention. First, although none of the participants were taking psychotropic medication at the time of scanning, a few of them were on medication for hypertension, diabetes, and heart disease. Therefore, the potential influences that these drugs had on resting-state connectivity could not be excluded. Second, the number of experimental subjects was relatively small. A larger independent data set would be required to validate the present findings, especially for determining ROI sensitivity/specificity, using the cut-off ReHo values reported here. Third, although individuals with StD are at a higher risk of developing MDD, we could not conclude that the observed brain activity alterations found in our StD subjects would also be found in clinical depression. To directly answer this question, an additional clinical depression group would be required to compare with the StD subjects. Better still, a longitudinal study design, with a large subject sample, would enable comparison of brain activity alterations that occur from normal state to subthreshold depression, all the way to clinical depression. Finally, we note that the abnormal activity observed in some brain regions of the StD subjects only demonstrated regional synchronization changes associated with spontaneous activity, and that the functional connectivity, as well as the global complex brain network properties of resting-state neural activity, remains to be addressed in future works.
